# Optical genome mapping identifies a balanced inversion disrupting *DMD* in a patient with Duchenne muscular dystrophy

**DOI:** 10.1186/s13039-025-00743-2

**Published:** 2025-12-07

**Authors:** Tuuni Turtinen, Pirjo Isohanni, Anna-Kaisa Anttonen, Leena Huhti, Katri Pylkäs, Marketta Tikkanen, Anna H. Hakonen, Sonja Strang-Karlsson, Tuomo Mantere

**Affiliations:** 1 Laboratory of Cancer Genetics and Tumor Biology, Translational Medicine Research Unit, Medical Research Center Oulu and Biocenter Oulu, University of Oulu, Oulu, Finland; 2 Department of Pediatric Neurology, Pediatric Research Center, Children’s Hospital, University of Helsinki and Helsinki University Hospital, Helsinki, Finland; 3 Stem Cells and Metabolism Research Program, Faculty of Medicine, University of Helsinki, Helsinki, Finland; 4 Laboratory of Genetics, Department of Clinical Genetics, HUS Diagnostic Center, Helsinki University Hospital, Helsinki, Finland; 5 Department of Medical and Clinical Genetics, University of Helsinki, Helsinki, Finland; 6 Department of Clinical Genetics, Fimlab Laboratories, Tampere, Finland; 7 Northern Finland Laboratory Centre, Oulu, Finland; 8 Department of Pediatric Neurology, Seinäjoki Central Hospital, Seinäjoki, Finland; 9 Department of Clinical Genetics, HUS Diagnostic Center, Helsinki University Hospital and University of Helsinki, Helsinki, Finland; 10 Faculty of Medicine, University of Helsinki, Helsinki, Finland

**Keywords:** Duchenne muscular dystrophy, Genetic testing, Karyotyping, Optical genome mapping, Chromosomal inversion

## Abstract

**Background:**

Duchenne muscular dystrophy (DMD) is a severe disorder that primarily affects males due to its X-linked recessive inheritance. It is caused by pathogenic variants of the *DMD* gene, most commonly exonic deletions, duplications, or point mutations. Current routine genetic testing methods, including next-generation sequencing and multiplex ligation-dependent probe amplification, can identify pathogenic *DMD* variants in over 90% of clinically diagnosed patients. However, in rare cases, a molecular diagnosis cannot be established using routine methods.

**Case presentation:**

We describe a follow-up genetic analysis, based on karyotyping and optical genome mapping (OGM), of a patient with clinically diagnosed DMD who initially had negative results in extensive routine genetic testing. Karyotyping revealed a paracentric X-chromosomal inversion with estimated breakpoints at p22.31 and p21.2. OGM fine-mapped this alteration as inv(X)(p22.2p21.1) and confirmed its pathogenicity by identifying the proximal breakpoint within intron 41 of *DMD*, thereby disrupting the gene and providing a definitive molecular genetic diagnosis.

**Conclusions:**

Current results further underscore the important role of chromosomal inversions as causal in a subset of DMD patients who remain without a molecular diagnosis after routine testing. It also demonstrates the utility of OGM in providing detailed, gene-level insights into cytogenetic abnormalities observed in the diagnostics of neuromuscular disorders.

## Background

Duchenne muscular dystrophy (DMD) is a severe form of muscular dystrophy characterized primarily by progressive skeletal and cardiac muscle degeneration due to lack of dystrophin protein in muscles [1]. About one-third of patients also exhibit intellectual disability [2]. DMD is inherited in an X-linked recessive manner and caused by pathogenic variants in the *DMD* gene, located on the short arm of the X chromosome (p21.2–p21.1). The spectrum of pathogenic *DMD* variants consists mainly of copy number variants (CNVs), including multi-exonic deletions (~60–70%) and duplications (~5–15%), as well as single nucleotide variants (SNVs) or small insertions and deletions (indels) (~20%) [1, 3, 4]. Deep intronic variants causing splicing defects and complex gene-disrupting structural variants (SVs) such as translocations and inversions are very rare (~1–2%) [3, 4]. Pathogenic *DMD* variants are identified in over 90% of clinically diagnosed patients using current routine genetic testing, which primarily consists of next-generation sequencing (NGS) and multiplex ligation-dependent probe amplification (MLPA) analysis [4, 5]. However, a small subset of patients remain without confirmed genetic diagnosis after these tests, likely due to deep-intronic variants or complex SVs [4, 6]. This highlights the importance of additional genetic testing that can detect alterations that are missed by conventional diagnostic methods.

Here, we describe a follow-up genetic analysis of a patient with clinically diagnosed DMD who remained genetically unsolved after extensive diagnostic testing. We applied optical genome mapping (OGM), a novel high-resolution technique for SV detection [7], with the aim of achieving molecular confirmation of the diagnosis and expanding knowledge of cryptic SVs affecting *DMD* that may escape the detection by routine analysis methods.

### Case presentation

The proband was a 3-year-old boy, the first-born child of the family, when he came to pediatric neurologic evaluation because of developmental delay. He had learned to walk independently at the age of 2 years, and his habitus was hypotonic. His creatine kinase (CK) was elevated up to 25 478 U/l, which together with motor delay, clumsiness, positive Gowers´ sign and mild calf hypertrophy raised suspicion of DMD. He also had a delay in cognitive and language skills and social interaction. His mother had an elevated CK level of 2 135 U/l. She reported some muscle stiffness in her legs and a history of learning difficulties at school. The proband’s maternal uncle was unaffected and none of the family members had a diagnosis of a neuromuscular disease.

### Genetic analysis

**Fig. 1:**
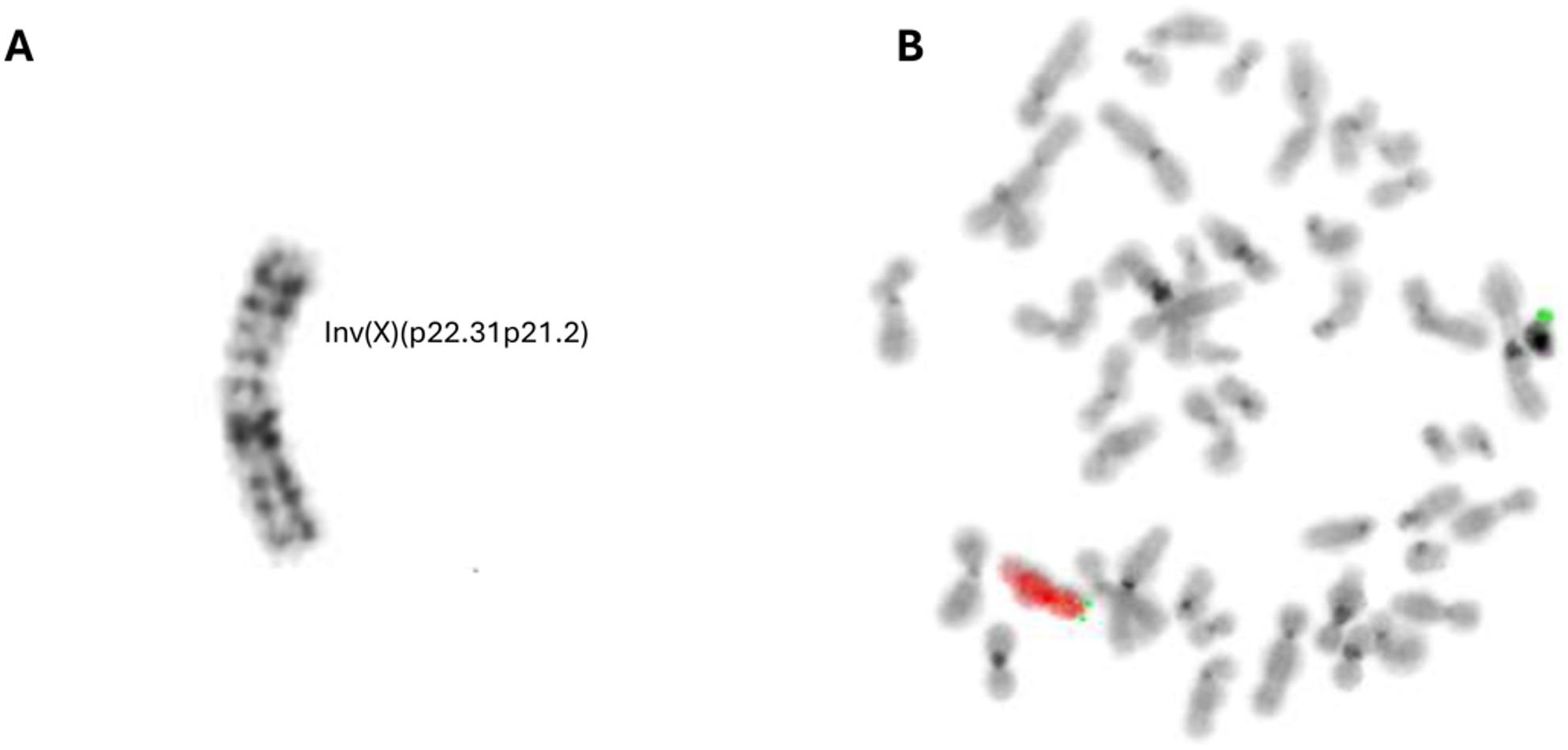
Identification of a paracentric X-chromosomal inversion. **(A)** G-banding analysis of the proband’s karyotype revealed a paracentric inversion with estimated breakpoints at p22.31 and p21.2. **(B)** FISH-analysis with whole chromosome X painting (red) and X and Y chromosome p-arm telomere region probes (green) ruled out the presence of X-chromosomal translocations

Following the initial clinical suspicion of DMD, MLPA analysis of the *DMD* gene was performed to detect exonic CNVs. Following negative MLPA results, extensive genetic testing was carried out, including (1) targeted NGS of the *DMD* gene, (2) fragile-X testing, (3) SNP-based CGH array, and (4) trio-exome sequencing of the index patient and parents, all of which were negative. Additionally, a chromosome G-banding analysis was performed, which revealed a seemingly balanced inversion with estimated breakpoints at p22.31 and at p21.2 (Fig. 1A). A fluorescence in situ hybridization (FISH) analysis by X-chromosome painting was performed to rule out the presence of translocations involving X-chromosome (Fig. 1B). Subsequent chromosome G-banding analysis confirmed that the mother of the index patient was a carrier of the same inversion.

**Fig. 2:**
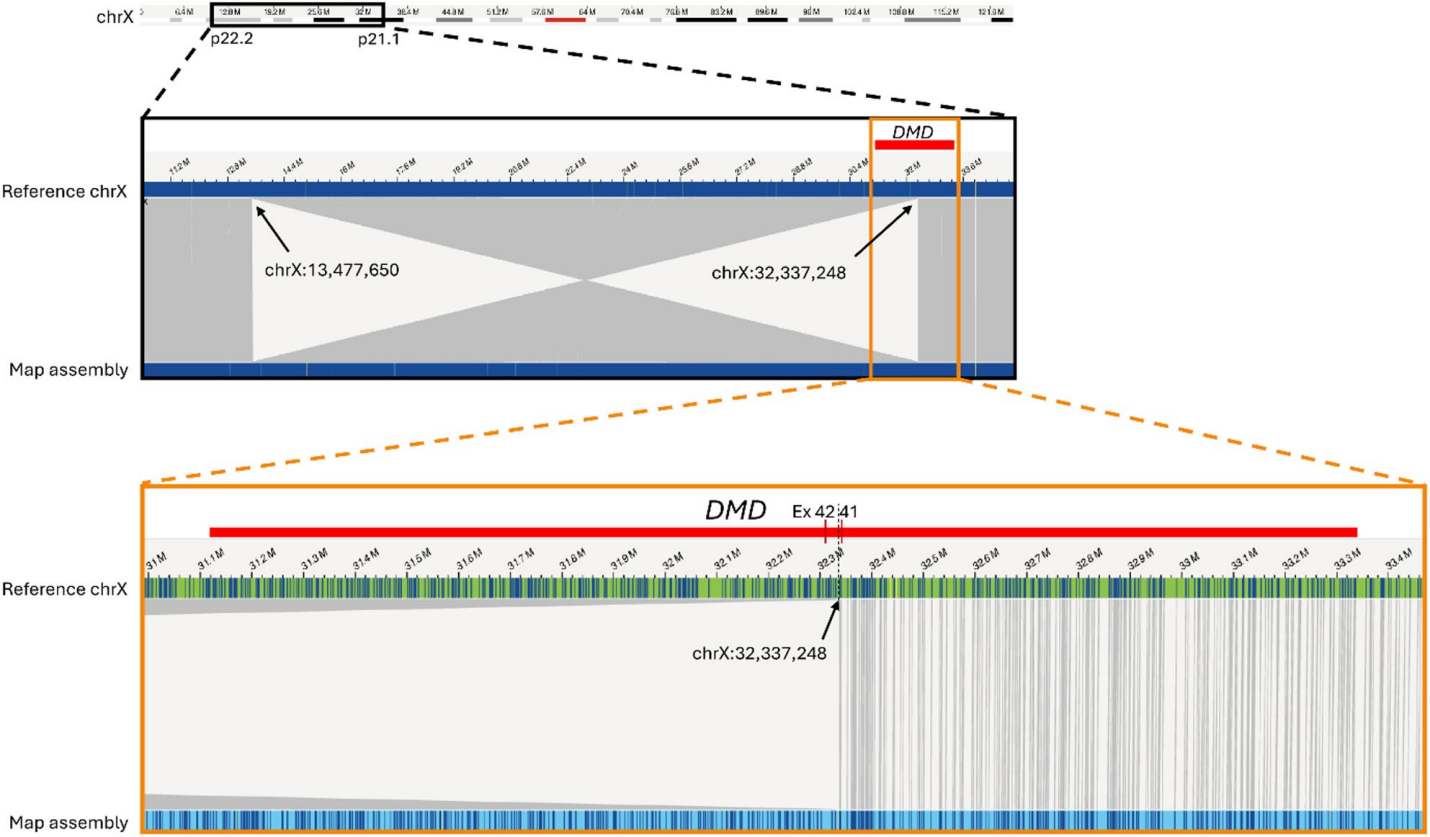
Optical genome mapping analysis of the index patient’s X-chromosome p-arm revealed a balanced inversion and its breakpoints at high-resolution, characterized as inv(X)(p22.2p21.1). The proximal breakpoint was located at chrX:32,330,404~32,337,248 (Hg38/GRCh38), residing within intron 41 of *DMD* (NM_004006.3). The distal breakpoint was located at an intergenic region chrX:13,447,650 ~ 13,483,855. Tilde (~) represents uncertainty of the exact breakpoint positions in the OGM analysis

**Fig. 3:**
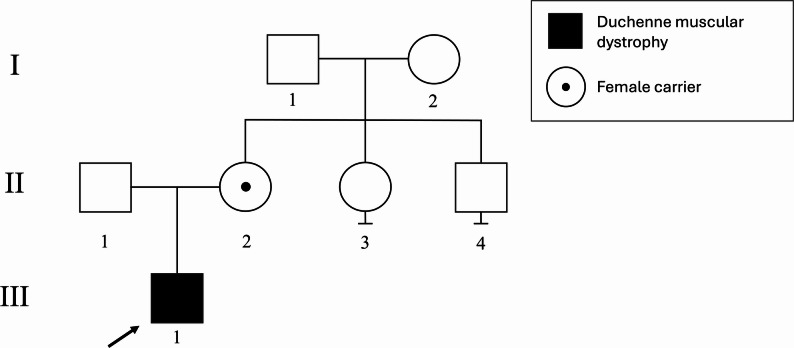
Pedigree. The proband (indicated by an arrow) was diagnosed at age 3 years. The carrier mother (II-2) had elevated creatine kinase level (2135 U/L) and reported learning difficulties in childhood and mild muscular symptoms. The maternal grandmother (I-2) is a confirmed non-carrier. The maternal uncle (II-4) and maternal grandfather (I-1) are asymptomatic adults and did not undergo carrier testing. The maternal aunt (II-3) is asymptomatic at adult age and was offered carrier testing with karyotyping and OGM due to the risk of parental germline mosaicism, but the results are still pending

Although the estimated proximal breakpoint of the inversion resided in the same cytoband as 3’ end of *DMD* (p21.2), karyotyping could not conclusively confirm its disruption. Therefore, to achieve high-resolution characterization of the inversion breakpoints, OGM analysis was performed as described previously [8]. This refined the inversion as inv(X)(p22.2p21.1) with proximal breakpoint located within intron 41 of *DMD*. The size of the inversion was 18.9 Mbp and the distal breakpoint resided within an intergenic region at p22.2 (Fig. 2). These findings provided a molecular diagnosis for the patient and enabled subsequent carriership testing in the family, showing that the maternal grandmother of the index was not a carrier of the inversion (Fig. 3).

Based on our findings, we reviewed the literature for similar reported cases by performing a proximity search using terms “DMD inversion“[Title/Abstract:~25] in PubMed and “DMD inversion” [all:~25] in PubMed Central. This search identified several studies published over the past decade in which balanced *DMD* inversions were detected using long-read technologies, including long-read sequencing and OGM, after routine genetic tests yielded negative results (summarized in Table 1) [9–25]. 

**Table 1:** Balanced inversions reported over the past decade in DMD cases with negative routine testing

Ref.	Year	Inversion	Size	Breakpoint 1	Breakpoint 2	Detection method
Current	2025	inv(X)(p22.2p21.1)	18.9 Mb	Intergenic	*DMD* intron 41	KT/OGM
[23]	2025	inv(X)(p22.11p21.1)	8.7 Mb	Intergenic	*DMD* intron 44	OGM/LRS
[23]	2025	inv(X)(p22.13p21.1)	14.7 Mb	*NHS*	*DMD* intron 44	LRS
[9]	2025	inv(X)(p21.1q26.2)	99 Mb	*DMD* intron 44	Intergenic	OGM
[10]	2024	inv(X)(p21.1q21.1)	50.7 Mb	*DMD* intron 55	Intergenic	srWGS
[11]	2024	inv(X)(p21.1q27.3)	110.8 Mb	*DMD* intron 47	Intergenic	LRS
[11]	2024	inv(X)(p21.3p21.1)	6.3 Mb	Intergenic	*DMD* intron 41	LRS
[12]	2024	Intragenic inversion	77 kb	*DMD* intron 67	*DMD* intron 62	LRS
[13]	2024	Intragenic inversion	297 kb	*DMD* intron 5	*DMD* intron 1	LRS
[20]	2023	inv(X)(p21.2p21.1)	1.3 Mb	Intergenic	*DMD* intron 45	OGM/LRS
[14]	2023	Intragenic inversion	216 kb	*DMD* intron 1	Intergenic	srWGS/LRS
[21]	2023	inv(X)(p21.1q21.31)	55 Mb	*DMD* intron 2	Intergenic	OGM/LRS
[21]	2023	inv(X)(p21.1q25)	95 Mb	*DMD* intron 16	Intergenic	LRS
[15]	2023	inv(X)(p21.1q26.2)	99.8 Mb	*DMD* intron 47	*HS6ST2*	KT/FISH/LRS
[16]	2022	inv(X)(p21.3p21.1)	5.9 Mb	Intergenic	*DMD* intron 2	LRS
[18]	2022	inv(X)(p22.12p21.1)	10 Mb	Intergenic	*DMD* intron 2	srWGS
[18]	2022	inv(X)(p22.2p21.1)	15 Mb	*GRPR*	*DMD* intron 54	srWGS
[24]	2022	Inv(X)(p22.31p21.2)	23 Mb	*VCX2-VCX3B*	*DMD* intron 60	RNAseq
[17]	2022	inv(X)(p21.1q13.3)	43 Mb	*DMD* intron 44	Intergenic	srWGS/KT
[19]	2020	Intragenic inversion	982 kb	*DMD* intron 51	*DMD* intron 7	LRS
[22]	2017	inv(X)(p21.3p21.1)	5.1 Mb	*ENSG00000228933*	*DMD* intron 38	srWGS/OGM
[25]	2017	inv(X)(p21.2q28)	119.8 Mb	*DMD* intron 17	Intergenic	RNAseq/srWGS
[25]	2017	inv(X)(21.1)	2.7 Mb	*DMD* intron 18	Intergenic	RNAseq/srWGS

FISH; fluorescence in situ hybridization, KT; karyotyping, LRS; long-read sequencing, OGM; optical genome mapping, srWGS; short-read whole-genome sequencing

## Discussion and conclusions

A wide spectrum of pathogenic *DMD* variants have been reported, most frequently exonic deletions, followed by duplications, point mutations and small indels [1]. In the majority of DMD cases, these variants are detected using routine diagnostic methods, generally MLPA analysis and/or NGS [4]. However, a small fraction of cases harbor genomic rearrangements, such as balanced inversions, that escape these standard methods, leaving these patients without a definite genetic diagnosis [4, 6]. 

In this case report, we describe a 3-year-old patient with strong clinical suspicion of DMD in whom standard genetic testing was negative, but karyotyping and subsequent OGM revealed a paracentric X-chromosomal inversion breakpoint disrupting DMD at intron 41. This finding provided molecular confirmation of the clinical diagnosis, which is important as it enables subsequent carrier testing as well as prenatal and preimplantation genetic testing in the family. Further testing of the maternal grandmother indicated that the inversion had likely arisen as a *de novo* in the patient’s mother, an important finding for appropriate genetic counseling of the family and determining the recurrency risk in future pregnancies.

With the advent of long-read technologies, pathogenic inversions disrupting *DMD* are being detected and reported more frequently. Considerable variation exists in inversion sizes and breakpoints; however, they all prevent production of the full-length primary muscle dystrophin isoform (Dp427m). Of the previously reported inversions causative for DMD, one additional patient harbored an inversion with a breakpoint located at intron 41 [11], similar to the patient described in the current report. Curiously, despite similar breakpoints within *DMD*, a neurodevelopmental deficit was present in our patient but not in the patient reported by Chen et al. As neither case involved disruption of additional genes at the inversion breakpoint outside *DMD*, the different genomic context into which *DMD* exons 42–79 are relocated may play a role. In particular, this altered context may lead to differences in the expression of *DMD* isoforms that could be transcribed from the relocated part of the gene, such as Dp140, which has been proposed to be important for neurodevelopment [2]. In our case, the exons 42–79 are relocated to a transcriptionally silent intergenic region within Xp22.2, which may prevent the transcription of *DMD* isoforms that have been implicated in cognitive and learning impairments. Although other unknown factors may contribute, these findings suggest that in cases of balanced inversions, both rearrangement breakpoints may play an important role in determining the phenotypic differences observed in DMD, warranting further investigations.

Considering genomic technologies, as suggested by Dremsek et al. and supported by this report, OGM is particularly valuable method in clinical scenarios where the analysis can be targeted to reveal SVs in certain disease-causing genes [26]. Although OGM does not provide nucleotide-level breakpoint information or detect SVs < 500 bp, its resolution is sufficient to confirm a clinical diagnosis in defined cases [26]. Mutation-negative DMD cases are a particularly well-suited target group to benefit from OGM analysis, given its monogenic nature and OGM’s ability to detect balanced abnormalities as well as other complex rearrangements that, although rare, are causal for the disease [9, 20–22, 27, 28]. Furthermore, in mutation-negative cases, muscle biopsy is often performed to assess dystrophin expression, with the absence of dystrophin supporting the clinical diagnosis of DMD. Improved genetic analysis could provide a less invasive means of confirming the diagnosis, since the detection of a pathogenic *DMD* variant would make muscle biopsy unnecessary [29], as in the case of our patient.

In conclusion, our study highlights the utility of OGM as an efficient method for detecting and refining cytogenetic abnormalities at high resolution. Balanced abnormalities, such as inversions, are often among the most difficult SVs to detect with short-read sequencing. However, a growing body of evidence shows that OGM, alongside long-read sequencing techniques, is an efficient method for detecting this SV type and the analysis is not confounded by the repetitive sequences that frequently mediate the formation of inversions. Altogether, the current report underscores the role of pathogenic inversions in *DMD* patients with negative genetic test results, provides insights into their association with intellectual disability, and supports the use of alternative long-read technologies in mutation-negative cases.

## Data Availability

Upon reasonable request.

## Abbreviations

CK: Creatine kinase
CNV: Copy number variant
DMD: Duchenne muscular dystrophy
FISH: Fluorescence in situ hybridization
indel: Insertion-deletion
MLPA: Multiplex ligation-dependent probe amplification
NGS: Next-generation sequencing
OGM: Optical genome mapping
SNV: Single nucleotide variant
SV: Structural variant
